# Sunstroke1Read at the meeting of the Pennsylvania State Veterinary Medical Association, Philadelphia, March 3, 1897.

**Published:** 1897-04

**Authors:** L. O. Lusson

**Affiliations:** Ardmore, Pa.


### SUNSTROKE.[1]

By L. O. Lusson, V.M.D.,
ARDMORE, PA.

Sunstroke is a sudden cerebral disturbance of an apoplectic nature due to exposure to excessive heat of the sun, occurring when there is an absence of humidity, and accompanied by delirium, coma, and death. Predisposing causes—plethora and disordered digestion, with torpidity of the liver.

*Direct Causes.* Exposure to the rays of a blazing sun.

*Symptoms.* If the driver is observing, he will notice that the animal is dull, that he does not sweat; his breathing is heavy, nostrils dilated; later his gait is unsteady, and a little later may stagger and fall, but will try to regain his feet and continue to rise and fall until, from exhaustion, he is compelled to remain down. He will throw the head up and down with great violence, and is unconscious of pain when striking the ground. If we make our examination at this time we find the skin extremely hot; mouth dry and very hot; mucous membrane congested; pulse rapid and cordy;

[1] Read at the meeting of the Pennsylvania State Veterinary Medical Association, Philadelphia, March 3, 1897.

respiration very fast; temperature from 105° to 107° F. This period of delirium may last from one to three hours. Then comes the period of collapse, the temperature suddenly falls two to four degrees, the surface becomes cold and clammy, the respirations are rapid, shallow, stertorous breathing, pulse rapid and feeble. The animal is then in a perfect state of coma, and if not treated usually dies in about an hour. The alterations are those of an intense cerebral congestion, the centres of the medulla most affected.

*Diagnosis.* From the symptoms and differentiated form of exhaustion, prodromes of laminitis, by the delirium, high temperature, and absence of diarrhœa.

*Prognosis.* Very fatal, on account of the inability to handle the subject.

*Treatment.* Perhaps bleeding in plethoric animals and at the beginning of delirium. Ice-packs or cold water over the surface. Ice to the head and in the mouth until temperature begins to fall; then push alcoholic stimulants concentrated in small quantities every half-hour. Application of alcohol with friction over the surface; warm clothing; hypodermic injection of strychnine, one grain every half-hour, when collapse is expected or subject does not rally under alcohol.

As this brief paper is only the result of my personal experience with sunstroke, and is written solely to bring forth a discussion, I ask for the kind indulgence of this Association.

